# Exploring psychological distress in Tunisian patients with laryngeal cancer after total laryngectomy

**DOI:** 10.1192/j.eurpsy.2025.1775

**Published:** 2025-08-26

**Authors:** L. S. Chaibi, A. Amri, O. Feki, M. Cheour, S. Hallit, F. Fekih-Romdhane

**Affiliations:** 1 Department of Psychiatry, Razi Hospital, Mannouba; 2 Head and Neck Carcinologic Surgery, Salah Azaiez Institute, Tunis; 3 Department of Psychiatry, University of Mannouba, Mannouba, Tunisia; 4 School of Medicine and Medical Sciences, Holy Spirit University of Kaslik, Jounieh, Lebanon

## Abstract

**Introduction:**

Approximately 450,000 new cases of head and neck cancers are diagnosed annually worldwide, with laryngeal cancer representing a significant proportion. In Tunisia, these cancers account for approximately 7.3% of all cancers diagnosed. Among those receiving surgical treatment, total laryngectomy is performed in nearly 50% of cases. Total laryngectomy (TL) is regarded as one of the most emotionally challenging surgical procedures due to its profound psychological and functional impacts.

**Objectives:**

This study aimed to investigate psychological distress (i.e. depression, anxiety, and stress) in Tunisian patients who had TL for laryngeal cancer.

**Methods:**

A descriptive cross-sectional study was conducted among 30 patients treated with TL in the Head and Neck Carcinologic Surgery Department at Salah Azaiez Institute.The operations took place during the period 2019-2022.Socio-demographic and clinical data were gathered. All patients completed the Arabic version of Depression, Anxiety and Stress scale (DASS-21).

**Results:**

The study involved 30 male participants with a mean age of 62 years (±10 years). All patients were married and tobacco users, and 53% lived in urban areas. Regarding the Tumor, Node and Metastasis (TNM) staging: 63% were classified as T3, 83% as N0, and 100% as M0. The therapeutic modalities include primary total laryngectomy (TL) followed by radiotherapy, an organ preservation protocol (OPP) followed by TL, partial laryngectomy (PL) followed by TL, and primary TL followed by both radiotherapy and chemotherapy.A total of 67%, 100% and 3% patients had moderate to severe levels of depression, anxiety, and stress respectively.In addition, our study found that a higher level of education was significantly associated with lower levels of depression and anxiety (P=0.001 and P= 0.012, respectively). Urban living was linked to reduced anxiety (P=0.05). Stage N1 was associated with higher depression levels (P=0.013). Finally, previous conservative treatments, such as partial laryngectomy or organ preservation protocol were associated with higher depression scores (P=0.001).

**Conclusions:**

Our findings showed high levels of psychological distress in patients with laryngeal cancer after total laryngectomy. Some factors correlated to distress in this group have been identified, enabling the identification of at-risk groups and effective prevention approaches.

**Disclosure of Interest:**

None Declared

